# Chronic vitamin D insufficiency impairs physical performance in C57BL/6J mice

**DOI:** 10.18632/aging.101471

**Published:** 2018-06-14

**Authors:** Kenneth L. Seldeen, Manhui Pang, Merced M. Leiker, Jonathan E. Bard, Maria Rodríguez-Gonzalez, Mireya Hernandez, Zachary Sheridan, Norma Nowak, Bruce R. Troen

**Affiliations:** 1 Division of Geriatrics and Palliative Medicine, Jacobs School of Medicine and Biomedical Sciences, University at Buffalo and Research Service, Veterans Affairs Western New York Healthcare System, Buffalo, NY 14203, USA; 2 New York State Center of Excellence in Bioinformatics and Life Sciences and Department of Biochemistry, School of Medicine and Biomedical Sciences, University at Buffalo, Buffalo, NY 14214, USA

**Keywords:** vitamin D, sarcopenia, muscle, mitochondria, microRNA

## Abstract

Vitamin D insufficiency (serum 25-OH vitamin D < 30 ng/ml) affects 70-80% of the general population, yet the long-term impacts on physical performance and the progression of sarcopenia are poorly understood. We therefore followed 6-month-old male C57BL/6J mice (*n*=6) consuming either sufficient (STD, 1000 IU) or insufficient (LOW, 125 IU) vitamin D3/kg chow for 12 months (equivalent to 20-30 human years). LOW supplemented mice exhibited a rapid decline of serum 25-OH vitamin D levels by two weeks that remained between 11-15 ng/mL for all time points thereafter. After 12 months LOW mice displayed worse grip endurance (34.6 ± 14.1 versus 147.5 ± 50.6 seconds, p=0.001), uphill sprint speed (16.0 ± 1.0 versus 21.8 ± 2.4 meters/min, p=0.0007), and stride length (4.4 ± 0.3 versus 5.1 ± 0.3, p=0.002). LOW mice also showed less lean body mass after 8 months (57.5% ± 5.1% versus 64.5% ± 4.0%, p=0.023), but not after 12 months of supplementation, as well as greater protein expression of atrophy pathway gene atrogin‑1. Additionally, microRNA sequencing revealed differential expression of mIR‑26a in muscle tissue of LOW mice. These data suggest chronic vitamin D insufficiency may be an important factor contributing to functional decline and sarcopenia.

## Introduction

Sarcopenia affects between 10% and 35% of individuals over the age of 65 and underlies functional decline and increases fall risk in older individuals [1,2]. Although the underlying causes of sarcopenia remain unknown, sarcopenia is associated with vitamin D insufficiency (25-OH vitamin D >10 ng/ml and < 30 ng/ml) [3,4]. Vitamin D insufficiency is also prevalent worldwide, affecting as many as 70% of the population across all age demographics [5,6], raising the likelihood that some individuals may be vitamin D insufficient for decades or more.

Vitamin D insufficiency has been associated with a range of physical performance impairments across multiple studies, including: poor grip and leg strength, lower scores in the short physical performance battery (SPPB), low activities of daily living (ADL) scores, and reduced physical activity in community dwelling older individuals [3,7,8]. Low serum 25-OH vitamin D (< 12.5 ng/ml) was also associated with worse physical performance as a composite score of gait speed, chair-stand, and grip strength in the Women’s Health Initiative (WHI) study [9]. Low serum vitamin D (<12.0 ng/ml) has also been associated with poor physical performance, fracture risk, and fractures in the Longitudinal Aging Study Amsterdam (LASA) [10]. However, several studies involving older individuals have reported no association between vitamin D and physical performance [11-13], raising uncertainty regarding the role of vitamin D in physical performance.

Animal studies can provide insights free from genetic and lifestyle factors that might confound the interpretation of human studies, leading to conflicting reports. Vitamin D receptor (VDR) knockout mice exhibit declines in several aspects of performance including swimming endurance, rotarod, and open field activity [14-16]. Furthermore, VDR ablation in muscle stem cells has demonstrated its critical role in muscle metabolism including muscle anabolic signaling [17,18], satellite cell proliferation [19], and regulation of the uptake of 25-OH vitamin D into muscle cells [20]. Recently a study silencing VDR expression with siRNA in muscle stem cells (C2C12) demonstrated inhibition of myogenic differentiation [21]. Dietary elimination of vitamin D has also been reported to impair performance, including lesser grip strength in mice [22] and swim endurance [23], while 1α-OH vitamin D treatment in ovariectomized rats was associated with increases in muscle strength, but not with muscle fatigue [24].

Animal studies rarely include the physiological relevant condition of vitamin D insufficiency, opting for vitamin D receptor knockouts or complete dietary elimination. Furthermore, there are no rodent or human studies that follow the impacts of vitamin D insufficiency for long periods of time (≥1 year for mice or about 20-30 years for humans), particularly while investigating physical performance. Therefore, we established groups of mice at serum vitamin D sufficient and insufficient levels, and followed changes in body composition and physical performance over a 12‑month period. Our findings demonstrate that vitamin D insufficiency modulates muscle miRNA signaling, increases atrophy pathway proteins, and impairs specific aspects of physical performance.

## RESULTS

### Depletion and repletion of serum 25-OH vitamin D occurs rapidly in response to changes in the amount of vitamin D3 supplementation, and levels remain consistent relative to the amount of supplementation

**Figure 1:**
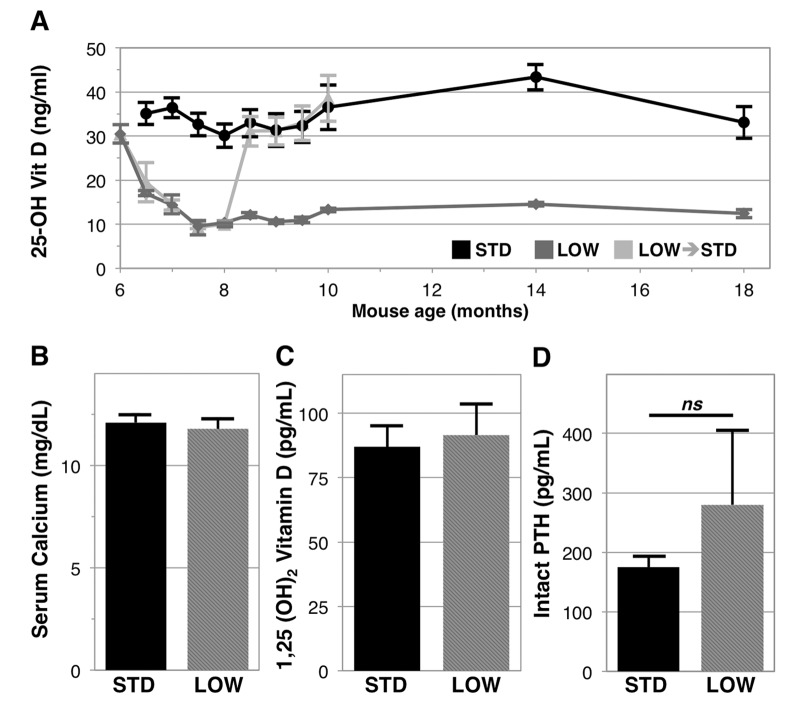
**Longitudinal analysis of 25-OH vitamin D in mice given differential vitamin D3 supplementation.** Six-month-old C57BL/6J male mice were given 1000 IU vitamin D_3_/kg chow (STD, black line, *n*=6), 125 IU (LOW, dark gray line, *n*=6), or were changed from 125 IU to 1000 IU after 2 months (light gray line, *n*=8) (**A**). After 12 months serum calcium (**B**), 1,25 (OH)_2_ vitamin D (**C**), and intact PTH (D), were assessed in STD and LOW mice using calorimetric assays or ELISA. Intact PTH was not significantly different (*ns, p=*0.10).

To understand the potential impacts of chronic vitamin D insufficiency we supplemented 6‑month old male C57BL6/J mice with standard (STD) facility levels of vitamin D3 in chow (1000 IU vitamin D3/kg) or reduced to a lower amount (LOW, 125 IU vitamin D3/kg) to induce vitamin D insufficiency. We observed that the STD level of supplementation results in stable serum 25‑OH vitamin D levels (30‑40 ng/ml) (Figure 1A, black line). We further observed that LOW supplementation leads to a rapid decline in serum 25‑OH vitamin D, reaching human equivalent levels of vitamin D insufficiency after just two weeks, and remaining consistently between 10-15 ng/ml for the remainder of the experiment (Figure 1A, dark gray line). After 4 months, we tested the rate of vitamin D repletion by switching a group of mice receiving 125 IU vitamin D3/kg to 1000 IU vitamin D3/kg chow (Figure 1A, light gray line), and found repletion also occurs within two weeks with resultant serum 25‑OH vitamin D levels in these mice similar to levels in the STD group. After 12 months, there were no statistically significant differences in serum calcium or levels of the active metabolite of vitamin D, serum 1,25‑(OH)_2_ vitamin D between the STD and LOW groups (Figure 1B & 1C, respectively). We did observe a trend towards higher serum intact parathyroid hormone in vitamin D insufficient mice (STD: 175.2 ± 18.4 pg/ml versus LOW: 279.7 ± 125.4 pg/ml, p=0.10, Figure 1D).

### Chronic vitamin D insufficiency did not affect body weight, but accelerates the reduction in lean body mass and the increase in fat mass

**Figure 2:**
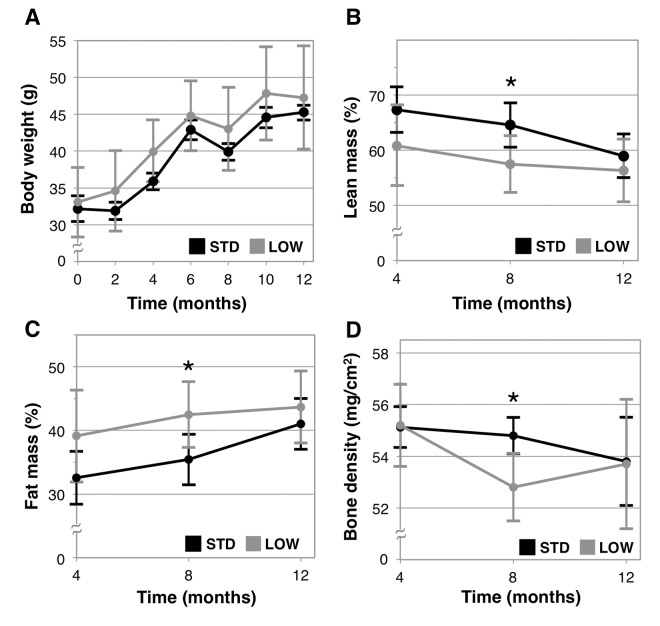
**Analysis of body composition and bone mineral density***.* Body weight was measured every two weeks in mice given standard (STD) and low (LOW) vitamin D_3_ in chow (**A**). Four months following the initiation of treatment lean mass (**B**), fat mass (**C**), and bone mineral density (**D**) were assessed using dual X-ray absorptiometry, *n=6* per group*,* “*” indicates p < 0.05.

We examined the impacts of chronic vitamin D insufficiency on body weight and composition. We found body weights to be similar between the two groups at all time points (Figure 2A), with equivalent overall weight gains (STD: 41.3% ± 10.6% versus LOW: 44.7% ± 15.6%, p=0.66), which is expected for mice of this age. Additionally, we examined lean body mass, fat mass, and bone mineral density using dual X-ray absorptiometry (DEXA). After 4 months of treatment (10 months of age), vitamin D insufficient mice trended towards a lower lean body mass and greater fat mass (p=0.08 for both, Figures 2B and 2C) and was significantly different from STD mice after 8 months (14 months of age, lean mass - STD: 64.5% ± 4.0% versus LOW: 57.5% ± 5.1%, p=0.0231 and fat mass - STD: 35.4% ± 4.0% versus LOW: 42.5% ± 5.2%, p=0.0243). However, there was no difference in either parameter after 12 months of treatment (18 months of age) (p=0.37). Bone mineral density of LOW mice was significantly lower than STD mice after 8 months (STD: 54.8 ± 0.7 mg/cm^2^ versus LOW: 52.8 ± 1.3 mg/cm^2^, p=0.0078, Figure 2D), but significant differences were not observed after 12 months.

### Vitamin D insufficiency impairs physical performance across multiple domains

**Figure 3:**
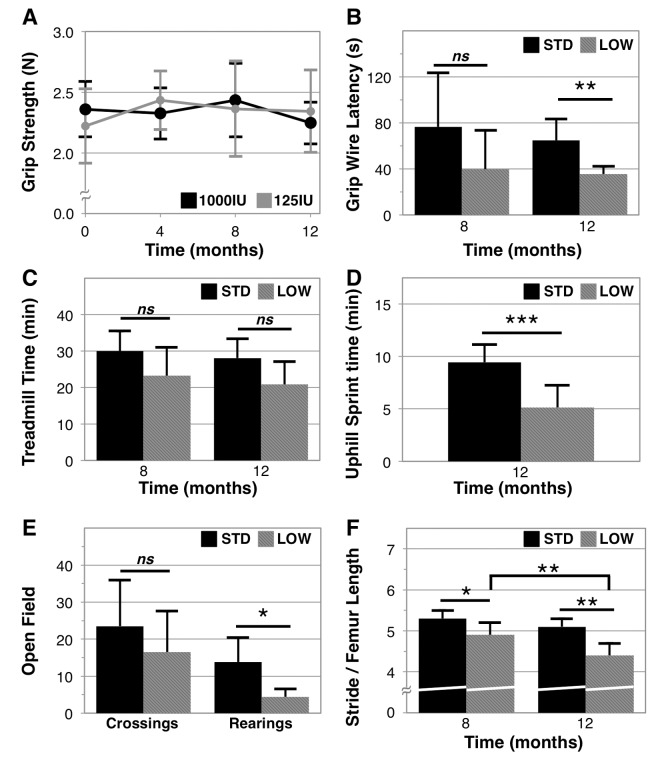
**Physical performance in vitamin D sufficient and insufficient mice***.* Vitamin D sufficient (STD) and insufficient (LOW) mice were assessed across a range of physical performance domains that include: grip strength assessed every 4 months as the best 3 of 5 trials on a grip strength meter, *n=*6 (**A**); grip endurance as the best of two trials timed for latency to fall from a wire, *n=5* (**B**); aerobic endurance assessed as a single trial for time before exhaustion on a mouse treadmill, *n*=6 (**C**); anaerobic endurance assessed as a single trial of increasing intensity intervals on an inclined (25º) mouse treadmill, *n=*6, 5 respectively (**D**); exploratory behavior as a count of quadrant crossings and rearings over 5 minutes in an open field arena, *n=*6, 5 respectively (**E**); and gait as assessed by measurement of stride length normalized to femur length determined using dual X-ray absorptiometry, *n=*5, 6 respectively (**F**). Statistical significance indicated by “*” p < 0.05, “**” p< 0.01, “***” p<0.001, and *“ns”* indicating non‑significance.

To examine whether vitamin D insufficiency impacts physical performance, and in what ways, we performed a range of behavioral assessments (Figure 3). We observed that grip strength did not significantly change over the 12‑month period of our experiment in either cohort (Figure 3A). There was also no difference in rotarod fall latency between supplementation groups, an assessment of balance and coordination, although both groups exhibited declines with age (STD baseline time to fall: 272.0 ± 45.4 seconds versus endpoint: 183.6 ± 39.1 seconds, paired T-test: p=0.014; LOW baseline: 231.7 ± 36.6 seconds versus endpoint: 157.8 ± 54.7 seconds, p=0.005, Figure S1A). However, vitamin D insufficient mice showed a deficiency in grip endurance, as determined by both grip wire (STD 65.8 ± 18.6 seconds versus LOW: 35.4 ± 6.7 seconds, *n =* 6 and 5, respectively, p=0.0039, Figure 3B) and grip grid latency (STD 147.5 ± 50.6 seconds versus LOW: 34.6 ± 14.1 seconds, *n =* 6 and 5, respectively, p=0.001, Figure S1B). No difference was observed in treadmill endurance (Figure 3C), although a difference between the two groups was trending (p=0.11 at 8 months and 0.06 at 12 months). The treadmill assessment was performed at 0˚ inclination, and gradual increases in speed likely imposed greater utilization of anaerobic respiration. However, to more completely examine if vitamin D insufficiency impairs anaerobic performance, we devised an uphill treadmill assessment by setting the inclination to 25˚ and introducing periods of active recovery at low speed to maximize anaerobic response. We observed that after 48 weeks, vitamin D sufficient mice achieved greater time before exhaustion than did vitamin D insufficient mice (STD: 9.4 ± 1.7 mins versus LOW: 5.1 ± 0.8 m/min, p=0.0007, *n =* 6 and 5 respectively, Figure 3D).

We further analyzed functional capacity by assessing open field activity. Although we did not observe a difference in open field exploration between the two groups (open field quadrant crossings: STD: 23.5 ± 12.4 versus LOW: 16.5 ± 11.0, p=0.33, *n =* 6 and 5, respectively, Figure 3E), we observed that vitamin D insufficient mice reared less often (STD: 13.8 ± 6.6 versus LOW: 4.4 ± 2.1, p=0.0141, *n =* 6 and 5, respectively, Figure 3E). Additionally, vitamin D insufficient mice exhibited significantly shorter stride length at 8 months (p=0.0318) and at 12 months (p=0.0018) (Figure 3F) and these mice also exhibited further decline in stride length from 8 to 12 months (LOW: 4.9 ± 0.3 to 4.4 ± 0.3 stride : femur length, p=0.0026, *n*=6), which was not observed in vitamin D sufficient mice (STD: 5.3 ± 0.2 to 5.1 ± 0.2, p=0.1757, *n*=5).

### Vitamin D insufficient mice exhibit trends for lower muscle fiber size and myofibrillar protein content, in addition to greater protein expression of atrophy associated atrogin-1

**Figure 4:**
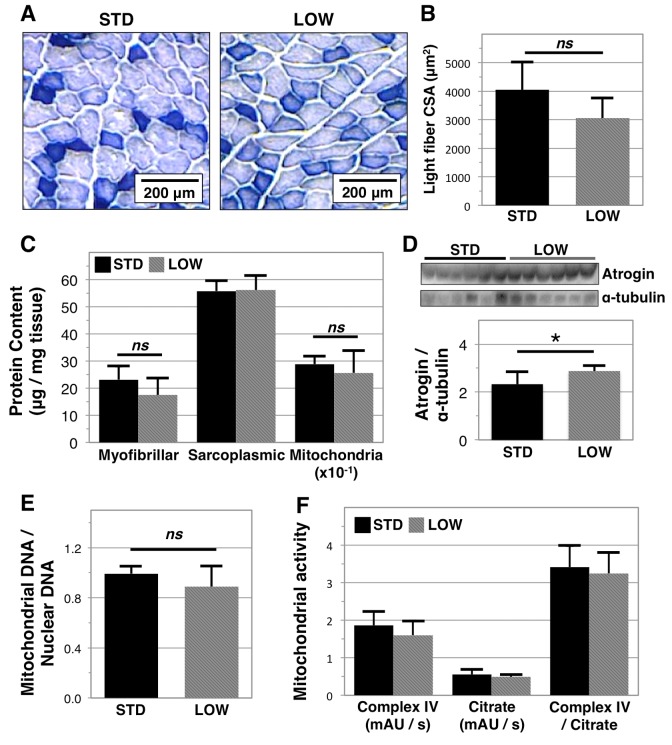
**Analysis of muscle quality in vitamin D sufficient and insufficient mice***.* To assess impacts of vitamin D insufficiency on muscle biology, tissues were harvested following 12 months of sufficient (STD) or insufficient (LOW) supplementation. Quadriceps muscle was then analyzed with NADH staining (**A**) allowing for quantification of the cross-sectional area (CSA) of light stain fibers that corresponds to fast twitch fibers, *n=*5, 4 respectively (**B**). Gastrocnemius muscle was analyzed by differential centrifugation to determine myofibrillar, sarcoplasmic, and mitochondrial protein content, *n=*6 (**C**). Atrogin-1 expression was also determined in gastrocnemius muscle with western blotting and relative expression (atrogin / tubulin) was quantified using ImageJ software, *n=*6 (**D**). Mitochondrial biomass (**E**) and activity (**F**) in soleus muscle, *n=*6, were determined by quantitative PCR and biochemical assays, respectively. Statistical significance indicated by “*” p < 0.05 and *“ns”* indicating non‑significance.

We next sought to identify factors that may be driving the observed differences in physical performance. NADH histological staining on quadriceps muscle (Figure 4A) showed a trend towards smaller cross- sectional area (CSA) in light stained fibers in vitamin D insufficient mice (LOW: 3,057.1 ± 708.3 versus STD: 4,046.7 ± 977.1 μm^2^ p=0.13, *n*=4 and 5, respectively*,* Figure 4B). We further examined if differences occurred in the content of specific muscle fractions by isolating myofibrillar, sarcoplasmic, and mitochondrial proteins using differential buffers and centrifugation (Figure 4C). We did not observe differences in sarcoplasmic protein content, yet there was a trend towards lower myofibrillar protein content in vitamin D insufficient mice (LOW: 17.4 ± 6.3 μg/mg tissue versus STD: 23.1 ± 4.9 μg/mg, p=0.11). Although these data are only suggestive, they are consistent with studies that report muscle decline in vitamin D receptor knockout models [25,26]. As aberrant vitamin D signaling is associated with greater atrophy pathway signaling [27], we investigated the expression of atrogin-1 and found greater expression in the quadriceps muscles of the vitamin D insufficient mice (STD: 2.33 ± 0.52 versus LOW: 2.88 ± 0.24, p=0.0393, Figure 4D). We did not observe differences in mitochondrial protein content (p=0.38, Figure 4C), the ratio of mitochondrial: nuclear DNA content in soleus muscle (STD: 0.99 ± 0.06 versus LOW: 0.89 ± 0.17, p=0.18, Figure 4E), or mitochondrial complex IV activity (p=0.82, Figure 4F).

### Vitamin D insufficient and sufficient mice exhibit a similar inflammatory profile

**Figure 5:**
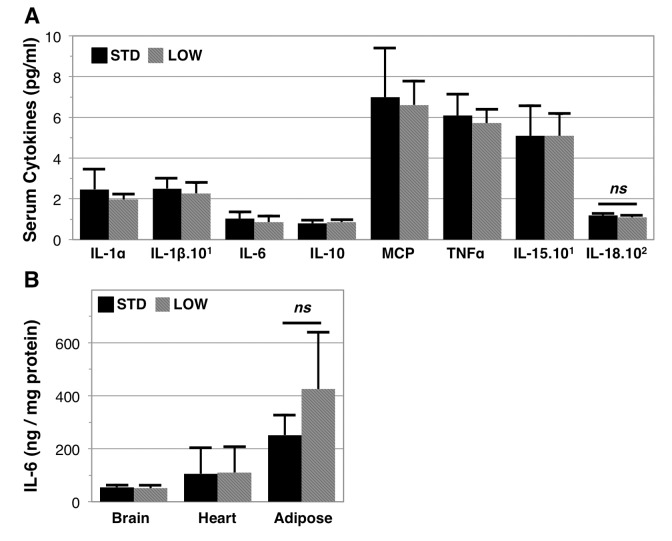
**Inflammatory profile of vitamin D sufficient and insufficient mice.** Serum at endpoint and used for multiplex ELISA analysis of serum cytokines, *n*=6 (**A**), *ns* denotes non-significance between vitamin D sufficient (STD) and insufficient (LOW) mice (IL-18: p=0.16). Interleukin-6 content was further assessed in brain, heart, and adipose tissues, *n=*6 (**B**), *ns* denotes non-significance in adipose IL-6 content (p=0.09).

Both clinical [28] and cell culture studies [29,30] support a role for vitamin D in modulating inflammatory cytokines. We therefore set out to determine if 12 months of vitamin D insufficiency modulated serum cytokine levels (Figure 5A, Table 1). Surprisingly, we did not identify any serum cytokine concentrations as being significantly different due to treatment, which included IL-1α, IL‑1β, IL-6, IL‑10, IL‑15, IL-18, MCP, and TNFα. We note that the serum level of IL‑18 was trending lower in vitamin D insufficient mice (STD: 118.0 ± 9.9 pg/ml versus LOW: 109.6 ± 9.2 pg/ml, p=0.16). We further investigated tissue concentrations of IL‑6 in brain, heart, and epididymal adipose tissue, but did not find any elevation in these tissues (Figure 5B). However, we also note that adipose tissue IL‑6 trended higher in vitamin D insufficient mice (STD: 251.7 ± 75.5 ng/μg protein versus LOW: 425.5 ± 213.6 ng/μg protein, p=0.09).

**Table 1:** Inflammatory cytokines in vitamin D sufficient and insufficient mice. Serum cytokines were determined after 12 months of STD or LOW vitamin D supplementation using multiplex ELISA.

**Cytokine**	**STD**	**LOW**	**p‑value**
**IL-1α**	2.5 ± 1.0	2.0 ± 0.3	0.27
**IL-1β**	25.1 ± 4.9	22.7 ± 5.3	0.44
**IL-6**	1.0 ± 0.3	0.9 ± 0.3	0.37
**IL-10**	0.8 ± 0.2	0.9 ± 0.1	0.44
**MCP**	7.0 ± 2.4	6.6 ± 1.2	0.73
**TNFα**	6.1 ± 1.0	5.7 ± 0.7	0.48
**IL-15**	50.9 ± 14.8	51.0 ± 10.8	0.99
**IL-18**	118.0 ± 9.9	109.6 ± 9.2	0.16

### Vitamin D insufficient mice exhibit a distinct muscle miRNA profile

**Figure 6:**
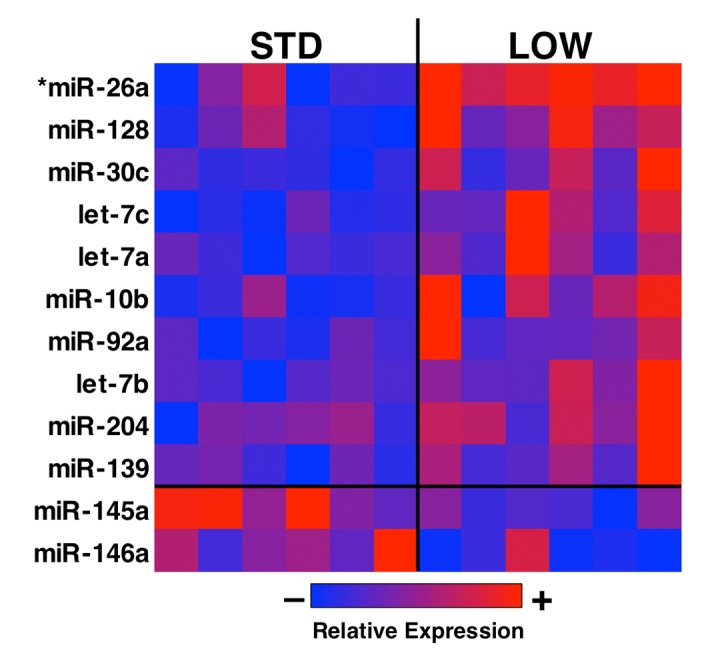
**Analysis of differential miRNA expression in vitamin D sufficient and insufficient mice.** Total RNA was isolated from tibialis anterior muscle and analyzed using miRNA sequencing. A total of 202 unique miRNAs reached thresholds for analysis, and a heat map was generated from 15 miRNA with a p<0.05 for differential expression between vitamin D sufficient (STD) and insufficient (LOW) mice (*n*=6). Further analysis for false positives (Q-val) revealed mir-26a as being differentially expressed between the two groups.

MicroRNAs are powerful effectors underlying many physiological processes, including the pathophysiology of muscle mass decline [31]. To investigate whether vitamin D insufficiency modulates muscle miRNA signaling, we isolated RNA from tibialis anterior muscles and then prepared samples for Next-Gen RNA sequencing using an Illumina NextSeq 500 high-throughput sequencing system. RNA sequencing revealed an average of 11.7 ± 1.5 million and 11.9 ± 1.4 million total reads per mouse for STD and LOW, respectively. Of these 4.8 ± 1.0 x 10^5^ and 4.9 ± 1.2 x 10^5^ reads for each STD and LOW, respectively, were mapped to known miRNAs using mIRBase v21. To avoid using insufficient data due to low expression, we removed any miRNA that did not reach at least 10 reads across 66% or more of the samples (per Jung et al. [32]), yielding a total of 202 unique miRNAs for further analysis. Our data revealed 12 miRNAs with potential for differential expression between STD and LOW (Figure 6 and Table 2, p<0.05); however, after false discovery rate correction we identified only one differentially expressed miRNA, mIR26a-5p, with a q‑value = 0.0392.

**Table 2:** Differential miRNA expression in vitamin D sufficient and insufficient mice.

**MicroRNA**	**mIRBASE ID#**	**Log_2_FC**	**P-value**	**Q-value**
miR-26a	MI0000573	0.07	0.000	0.04
miR-128	MI0000155	0.32	0.003	0.15
miR-30c	MI0000547	0.17	0.005	0.15
Let-7c	MI0000559	0.17	0.005	0.15
Let-7a	MI0000556	0.17	0.016	0.15
miR-10b	MI0000221	0.25	0.018	0.36
miR-92	MI0000719	0.11	0.025	0.41
Let-7b	MI0000558	0.55	0.030	0.44
miR-204	MI0000247	0.21	0.032	0.44
miR-139	MI0000693	0.21	0.048	0.56
miR-145a	MI0000169	-0.14	0.036	0.46
miR-146a	MI0000170	-0.56	0.021	0.38

## DISCUSSION

Vitamin D insufficiency is a prevalent condition for which the long-term impacts are poorly understood. Here we demonstrate that mice kept in a serum 25‑OH vitamin D insufficient state for 12 months exhibit significant declines across multiple domains of physical performance. These domains include grip hang endurance, uphill sprint, and open field rearing, which together may be indicative of a decline in anaerobic capacity due to vitamin D insufficiency. The loss of anaerobic capacity is partially supported by a trend (p=0.13) towards lower smaller fast twitch fiber CSA in vitamin D insufficient mice and lower lean mass in vitamin D insufficient mice, although this latter difference was of limited duration. Belenchia et al. [33] also observed loss of lean mass in mice due to dietary vitamin D deficiency (serum 25‑OH vitamin D < 10 ng/ml) of approximately 40 weeks in female mice initiated at 8 weeks of age. We suspect our study was underpowered to identify such histological differences; however, the possibility that vitamin D insufficient mice exhibit smaller fast twitch fiber CSA is supported by our finding that vitamin D insufficient mice also exhibit greater expression of atrogin-1, for which this and other atrophy associated proteins have previously been linked to vitamin D signaling [27,34,35]. Thus these data support the notion that long-term vitamin D insufficiency may contribute to the progression of sarcopenia. Our finding that vitamin D insufficient mice exhibit shorter stride lengths further emphasizes the potential contribution of vitamin D status, as such gait disturbances are an integral component of functional capacity decline associated with sarcopenia [36,37].

Other areas of physical performance were not impacted in the time frame of our study. Surprisingly, we did not identify differences in grip strength in our mice in contrast to multiple human studies reporting an association between vitamin D and strength [38-41], but not all [42,43]. However, we did observe a trend towards lower myofibrillar protein content (p=0.11), which may be indicative of future grip strength decline had we continued our experiment beyond 12 months. We also did not observe significance differences in treadmill performance, although vitamin D insufficient mice were trending towards lower treadmill performance at both 8 months (p=0.11) and 12 months (p=0.06). We believe this may be due to a greater anaerobic requirement as opposed to a possibility of aerobic deficit, as supported by our finding of differences when assessing treadmill performance using uphill intervals. Our findings that neither mitochondrial biomass nor activity were affected by vitamin D insufficiency further supports the idea that both groups exhibit similar aerobic capacity.

Additionally, although the relationship between vitamin D and fall risk is suggestive, it remains inconclusive [44]. We anticipated vitamin D insufficient mice would exhibit worse rotarod performance, as rotarod is an indicator of balance and coordination [45]. Yet our data show both groups exhibit age-dependent declines. These findings are surprising in light of Sakai et al. [46] who reported vitamin D receptor ablation impairs rotarod performance, perhaps indicating that stark vitamin D deficiency or receptor ablation is necessary or that such differences would have appeared if we continued the experiment into advanced ages (> 24 months of age).

We found long term impacts of serum 25‑OH vitamin D on body weight and body composition, which is consistent with our 6-month study examining the impacts of alterations of serum 25‑OH vitamin D levels in lean and obese mice [47]. However, Belenchia et al. reported declines in body weight in vitamin D deficient mice after 6 months until the endpoint of the study after 10 months, at which point these mice also exhibited decreased fat mass and lean body mass [33]. In contrast, our study identified a decrease in lean mass, and an increase in fat mass, after 8 months of insufficiency that was not observed after 12 months. However, the similarities and differences in our body composition findings compared to Belenchia et al. may be explained by the use of males versus females, insufficiency versus deficiency, and that our study continued two months longer. It was also surprising that our study failed to show differences in the inflammatory milieu in light of the reported relationships between serum vitamin D and chronic inflammation [28]. In particular, lower serum vitamin D was previously shown to be associated with increased IL‑6 expression [28,48,49], yet we did not observe any increases in serum, brain, or cardiac tissues. We did observe a trend of greater IL‑6 expression in adipose tissue of vitamin D insufficient mice (0.09), which would be consistent with mechanistic reports of the impacts of vitamin D in adipocytes [50].

Our analysis of miRNA sequencing revealed a single miRNA, miR-26, was differentially expressed in vitamin D insufficient mice. miR-26 has previously been shown to be important for skeletal muscle differentiation [51,52], and was found in two human studies to be differentially expressed in response to exercise [53,54]. Additionally, miR-26a was differentially expressed in two separate pilot studies that included 5 subjects supplemented with high dose vitamin D for a 12‑month period [55]. However, the authors further reported that mIR-26a was not differentially expressed in a larger scale study [55], indicating the need for additional studies to better elucidate the impact of vitamin D status on miRNA profiles and expression. Our study also identified other miRNAs with reported roles in skeletal muscle biology including miR-204 [56], miR-139 [57,58], miR-146 [58-60], and miR‑30 [58,61], although none of these met the threshold for false discovery rate (q < 0.05). We think this may be in part due to insufficient power, which may have also prevented us from identifying differences in other parameters (i.e. fiber size, inflammation, physical performance). Additionally, our low sequencing depth (of just under 500,000 mapped miRNA reads per sample) may have also restricted our ability to discern differences in only those miRNAs with high expression [62].

Our study also confirms the findings that altering vitamin D supplementation results in a rapid shift (both depletion and repletion) in serum 25-OH vitamin D levels (within 2 weeks) that is sustained relative to the amount of supplementation [33,47,63]. Our 12-month study was approximately 6 weeks longer than Belenchia et al. [33], which was performed in female mice, and together these studies demonstrate little to no impact of gender on the relationship between vitamin D supplementation and serum 25-OH vitamin D concentration. Interestingly, we did not observe differences in serum 1,25‑(OH)_2_ vitamin D between 25‑OH vitamin D sufficient and insufficient mice. Although this finding is consistent with our previous study when we induced vitamin D insufficiency for 6 months in mice [47], it remains surprising in light of the functional role of 1,25‑(OH)_2_ vitamin D in skeletal muscle regulation [64-66]. In particular, Hassan‑Smith et al. have reported that serum 1,25‑(OH)_2_ vitamin D concentrations correlated with leg power, velocity, and jump height in 20-74 year old males and females, parameters that were not found to be correlated with serum 25-OH vitamin D [67]. Serum 1,25‑(OH)_2_ vitamin D was also found to be correlated with low muscle mass in cross-sectional analyses of men and women aged 21-97 years, as well as knee extension force in women [11]. Additionally, both low serum 25‑OH and 1,25‑(OH)_2_ vitamin D were independently found to be associated with the incidence of sarcopenia at a 5 year follow-up in men >70 years of age [68]. Yet, Boonen et al. reported no association between 1,25‑(OH)_2_ vitamin D and knee extension strength in women aged 70 to 90 [69], and Gielen et al. also reported no association between 1,25‑(OH)_2_ vitamin D and physical performance, specifically grip strength and gait speed in men aged 70 and older [70]. These studies were observational and did not involve long-term abatement of dietary vitamin D3. It is therefore possible that the duration and the degree of serum 25-OH vitamin D reduction may affect outcomes. Another possibility is that serum measures of 1,25‑(OH)_2_ vitamin D in our year long study did not reflect the actual 1,25‑(OH)_2_ vitamin D within muscle since skeletal muscle expresses the 25‑hydroxyvitamin D3 1-alpha-hydroxylase [20,71].

With regards to other serum markers, Belenchia et al. reported changes in serum calcium levels in vitamin D deficient mice, which was not observed in this study, our previous study [47], or by Mallya et al. [63]. Likewise, Belenchia et al. was the only study to report significant differences in PTH concentrations; however, intact PTH was trending towards elevation in our vitamin D insufficient mice (p=0.10), and our study may have been insufficiently powered to observe this. Despite these differences, the behavior of serum 25-OH vitamin D in response to altered vitamin D supplementation was consistent between these studies and supports the use of the dietary supplementation and deprivation to examine serum 25-OH vitamin D related phenomena.

## CONCLUSION

Serum 25-OH vitamin D declines rapidly and remains consistently depressed in response to low supplementation. Prolonged vitamin D insufficiency induces characteristics of sarcopenia that include poor anaerobic capacity, lower lean mass, and a trend towards smaller fast twitch fiber CSA, as well as gait disturbance. Vitamin D insufficient mice also exhibited increased expression of atrophy-associated Atrogin-1 and differential expression of muscle regulation associated miR-26a. These data suggest a role for chronic vitamin D insufficiency in the development of sarcopenia, highlighting the need for further animal and human studies to investigate the impacts of vitamin D during aging.

## MATERIALS AND METHODS

### Animals

Twelve C57BL6/J mice (5 months old) were purchased from Jackson labs (Bar Harbor, ME). After 1 month the mice were randomly sorted into groups (*n=6)* that received AIN‑93G chow (Dyets Inc., Bethleham, PA) supplemented with either the standard facility amount of 1000 IU vitamin D3 / kg chow (STD) to maintain serum 25-OH vitamin D sufficiency or 125 IU vitamin D3 / kg chow (LOW) to induce serum vitamin D insufficiency over a period of 12 months (Table 3). Additionally, eight mice were initially supplemented with 125 IU, but then switched after two months to 1000 IU to examine the rate of serum 25-OH vitamin D repletion. Food and water were provided *ad libitum*, and mice were housed in large shoebox animal cages containing 6 or 8 mice per cage. Lighting was on a 12 hour on / 12 hour off cycle, and cages were shielded to reduce exposure to facility lighting. Body weight was measured every two weeks. All studies and experimental protocols were approved by and in compliance with guidelines of the Miami VA and VA Western New York Animal Care and Use Committees.

**Table 3:** Diet compositions.

**Component**	**Content (*per kg chow)***
Vitamin Free Casein	200 g
L-Cystine	3 g
Dyetrose	35 g
Sucrose	349 g
Cornstarch	3.6 g
Soybean oil	9 g
Lard	20 g
t-Butylhydroquinone (TBHQ)	0.005 g
Cellulose	50 g
Dicalcium Phosphate	13 g
Calcium Carbonate	5.5 g
Potassium Citrate	16.5 g
Choline Bitartrate	2 g
Vitamin/Mineral Mix *(Vit D free)*	20 g
Vitamin D_3_ (cholecalciferol)	STD: 1000 IU (25 μg) LOW: 125 IU (3.1μg)

### ELISA and Colorimetric assays

Blood was collected through the sub-mandibular vein using a mouse lancet (MEDIpoint, inc., Mineola, NY.) into microcentrifuge tubes. Samples were held at room temperature for 10 minutes to allow coagulation and then centrifuged at 16,0000 x g for 10 minutes at 4 °C to allow separation of serum. Analysis of serum was performed using ELISA kits for 25-OH vitamin D (ImmunoDiagnostic Systems, Inc., Scottsdale, AZ), 1,25-(OH)_2_ vitamin D (MyBioSource, San Diego, CA), and intact PTH (MyBioSource). Colorimetric assays were performed to assess serum calcium concentration (Biovision, San Francisco, CA.) according to manufacturer protocols. Multiplex ELISA was performed using a multi-analyte ELISA plate (Biorad, Hercules, CA) that includes IL-1α, IL‑1β, IL-6, IL-10, IL-15, IL-18, MCP, and TNFα, which was then analyzed using a Bio-plex Magpix (Biorad, Hercules, CA).

### Dual-energy X-Ray Absorptiometry (DEXA)

Analysis of bone mineral density, body fat % and lean mass were performed using a Lunar PIXImus II (GE Healthcare, United Kingdom). Animals were anesthetized using a ketamine/xylazine cocktail and then analyzed with a single scan after 4 months of treatment and every 4 months thereafter.

### Physical performance assessments

A single investigator, blinded to the group designations of the mice, performed all animal assessments. Additionally, all experiments were performed during lighted hours and at the same time of day at each assessment time point. Protocols for each assessment were as follows:

#### Grip strength

Maximal grip strength data was generated using a Columbus instruments grip force meter (Columbus, OH) as the average of the best 3 of 5 trials for each mouse. For each trial the mouse was held firmly near the base of the tail and placed with all four paws upon a metal grid attached to a force meter. The mouse was then pulled such that the body of the mouse was parallel to the ground until the mouse lost grip. Mice were given 10 seconds of rest between trials.

#### Treadmill endurance

The mice were given a single assessment at each time point on a Columbus instruments treadmill set with no inclination (flat - 0°). In the trial, the belt slowly accelerates from 5 to 25 m/min over 60 minutes and the mouse is timed until exhaustion, defined as 10 visits to a shock pad (54V, 0.72mA), 20 total shocks, or having remained on the treadmill belt for 60 minutes. Prior to the initial assessment, mice were given 3 similar trials (separated two weeks apart) to acclimate the animals to the device.

#### Uphill sprint assessment

To assess uphill sprint endurance the treadmill was inclined at 25° and mice were given a warm up period of 1 minute at 5 m/min. This was then followed by intervals that started at 10 m/min for 20 seconds and increased by 1 m/min increments after each 20 second active recovery period at 5 m/min. The mouse continued until exhaustion, defined as visiting the shock pad 5 times or receiving 10 total shocks.

#### Grip wire endurance

Mice were placed on a 5 mm thick neoprene wire and timed until fall. Dividers were placed on either side of the wire to prevent mice from leaving the apparatus. Mice were given three attempts to attain a minimum 15 seconds per trial, and the better score of two trials was used for each mouse.

#### Stride length

To measure stride length, the front paws of the mouse were coated in dye (Bradford reagent) and the mouse was then placed on one end of an apparatus (75 cm x 30 cm wide) that was lined with paper. A mouse shelter from the home cage was inserted on the other end of the apparatus to motivate the mouse to walk. Stride length was measured as the average of 5-6 steps per mouse as the distance between the centers of the paws. The stride length was further normalized by femur length as determined by measuring images of the femur generated by DEXA.

#### Open field activity

To assess spontaneous activity the mouse was placed into a 90 cm x 90 cm apparatus that was divided equally into 4 quadrants. An investigator was positioned approximately 3 feet away and manually counted crossings into new quadrants and rearings (standing on hind legs) over a 5‑minute period.

### Muscle histology

NADH histological analysis of quadriceps muscle (rectus femoris) was assessed as described previously [72]. Briefly, 10 µm frozen muscle sections were submerged in a solution containing 1 mg/ml NADH (Sigma, St. Louis, MO), 1 mg/ml Nitro Blue Tetrazolium (VWR #TCD0844), and 0.2 M Tris‑HCl buffer at pH 7.4 for 45 minutes at 37° C. Sections were then immersed in a series of acetone baths, rinsed in distilled water, and dehydrated by immersing in ethanol and xylene, before finally mounting on a cover slip with Cytoseal (Fisher #23-244257). An investigator, blinded to the identity of the mice, identified and tallied fiber types and also measured cross sectional area (CSA) of the fibers using Motic software (Motic, Hong Kong).

### Mitochondrial biomass

Total DNA was isolated from soleus and anterior tibialis muscle using a Qiagen Tissue Quick mini-prep kit (Qiagen, Germantown, MD). Primers were designed to amplify mitochondrial DNA (forward: 5’-CCGCAAGGGAAAGATGAAAGA-3’, reverse: 5’-TCGTTTGGTTTCGGGGTTTC-3’) and nuclear DNA (hexokinase gene, forward: 5’-CCCTGTCATGTCCCTTTGTT-3’, reverse: 5’‑GCCACCAGCTCAGTTAAAGG-3’) and then amplified using quantitative PCR (LightCycler 2.0, Roche).

### Mitochondrial activity

Mitochondrial activity was assessed as described previously [72]. Briefly, soleus muscle was homogenized in Chappel-Perry isolation medium (100 mM KCl, 50 mM Tris‑HCl, 5 mM MgCl_2_, 1 mM ATP, 1 mM EGTA, pH 7.5) and centrifuged 10 minutes at 900 x g. The supernatant was collected and centrifuged for 10 minutes at 9,000 x g at 4 °C, and the mitochondria rich pellet was then washed once and resuspended in ice‑cold SHE buffer (250 mM sucrose, 10 mM Hepes, 1 mM EGTA, pH 7.2). Protein concentration was determined using a Bradford assay. Complex IV activity was determined as the rate of absorbance (412 nm) decline induced by adding 1 μg of mitochondria into a cuvette containing 1 mL of ice‑cold reaction buffer (10 mM KH_2_PO_4_, 250 mM sucrose, 1 mg/ml BSA, 10 µM reduced cytochrome C (reduced using sodium hydrosulfite), 2.5 mM lauryl maltoside, pH 6.5). Citrate activity was determined as the rate of absorbance (412 nm) increase by adding 1 μg of mitochondria extract into a cuvette containing 500 μL of ice‑cold reaction buffer (100 mM Tris, 2 mM DTNB (5,5-dithio-bis-2-nitrobenzoic acid), 4 mM oxaloacetic acid, 1 mM acetyl Co-A, pH 8.0).

### Analysis of muscle myofibrillar, sarcoplasmic, and mitochondrial fractions

Our methodology to assess relative percentages of protein fractions was adapted from previous studies [73-75]. Approximately 30-40 mg of gastrocnemius muscle was homogenized using a pestle tissue homogenizer in 3.0 mL of ice-cold analysis buffer (20 mM tris-HCl, 250 mM sucrose, 100 mM KCl, 5 mM EDTA, pH 6.8). The homogenate was centrifuged at 1,000 x g for 15 minutes. To isolate the myofibrillar fraction, the pellet was washed twice with 5 mL of ice cold wash buffer (20 mM tris‑HCl, 175 mM KCl, 5 mM EDTA, 0.5% (v/v) Triton-X100 pH 6.8) and centrifuged at 1,000 x g for 10 minutes at 4°C. The pellet was suspended in 1.0 mL of ice‑cold analysis buffer for subsequent protein determination. To isolate the mitochondrial and sarcoplasmic fractions, the initial supernatant was centrifuged at 9,000 x g for 20 minutes at 4°C. The supernatant was then collected as the sarcoplasmic fraction for subsequent protein determination. The pellet was washed in 5 mL of ice cold SHE buffer (250 mM sucrose, 10 mM Hepes, 1 mM EGTA, pH 7.2) and centrifuged at 9,000 x g for 20 minutes at 4°C. The pellet was then suspended in 100 μL of ice‑cold SHE buffer. Determination of protein concentration was performed using a Bradford assay and used to determine the protein content of each fraction normalized to mg of wet tissue weight.

### Western blot analysis

Gastrocnemius muscle was homogenized in ice‑cold extraction buffer (20 mM HEPES, 1 mM EDTA, 5 mM EGTA, 1.5 mM NaVO_4_, 10 mM MgCl_2_, 50 mM glycerolphosphate, 2 mM DTT, 10 mM NaF, 1% Triton X-100, 100 µM PMSF, pH 7.4) and protein concentration was determined using Bradford assay. Proteins were resolved by protein electrophoresis by applying 30 μg of each sample onto a 10% Biorad gel (Ready Gel Tris-HCl -101-1394). Assessment of atrogin-1 expression was performed using overnight incubation of sample with antibodies against atrogin-1 (ThermoFisher Scientific, Cat# PA5-19056, Waltham, MA) and tubulin (ThermoFisher Scientific, Cat #62204) for normalization. Quantitation was performed using ImageJ software (NIH).

### MicroRNA analysis

Total RNA was harvested from tibialis anterior muscle using Qiagen miRNAeasy Purification Kit (Qiagen, Germantown, MD) according to the manufacturer’s instructions. RNA libraries for sequencing were established using the NEBNext Multiplex Small RNA Library preparation kit, and the MiRNA libraries were sequenced on the Illumina NextSeq 500 generating 76-cycle single reads. Demultiplexing was performed with Illumina’s bcl2fastq version 2.17.1.14. General sequence quality was evaluated with FastQC, and reads were trimmed of adapters using trim galore v0.4.4. Subsequently, reads were aligned to the Ensembl GRCm38 genome build using bowtie2 v2.2.8 with the very-sensitive-local parameter set [76]. Aligned reads were quantified using featureCounts [77] against miRBase v21 miRNA database, and the resulting counts were tested in R using the Bioconductor package DESeq2 [78]. MiRNA with counts of less than 10 reads in 66% of the samples, post normalization, were removed from the analysis. Statistical analysis was performed using DESeq2, which includes a Benjamini‑Hochberg correction for false positives [79].

### Statistics

Statistical analysis was performed using XLStat statistical software (Addinsoft, New York, NY). A Student’s *t*-test was used for all comparisons of standard (STD) supplementation versus insufficient (LOW) supplementation. All data were screened for outliers using a Grubbs outlier test with alpha equal to 0.05. The cut-off for significant comparisons was p < 0.05. All data are presented as mean ± standard deviation.

## Supplementary Material

Supplementary File
