# Supplementary material for: Chronic vitamin D insufficiency impairs physical performance in C57BL/6J mice
Source: Aging (Albany NY). 2018 Jun 14;10(6):1338–55. doi: 10.18632/aging.101471 (PMC6046224; doi:10.18632/aging.101471)
Supplement: Supplementary File [file aging-10-101471-s001.pdf]

SUPPLEMENTARY MATERIAL

SUPPLEMENTARY METHODS

Rotarod balance and coordination

Mice are timed for ability to stay atop a spinning cylinder that increases in speed from 4 to 40 RPM over 5 minutes. Latencies to fall or a maximum time of 360 seconds are recorded in 3 trials. The mice tolerate the fall without harm.

Grip Grid

Mice are placed on a wire grid and then inverted over a 30 cm x 30 cm x 45 cm (L x W x H) box and timed until fall. Three timed trials are administered, and the trial is stopped if the mouse falls or maintains a grip for 600 seconds. The mice tolerate the fall without harm.

SUPPLEMENTARY FIGURE

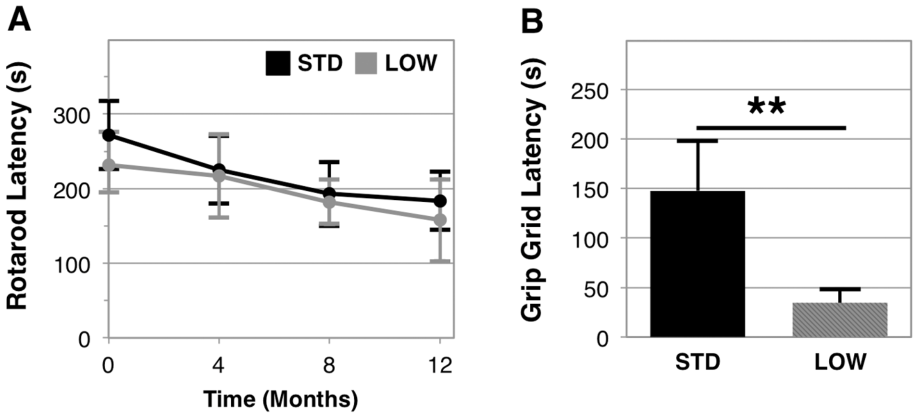

Figure S1. Physical performance in vitamin D sufficient and insufficient mice. Vitamin D sufficient (STD) and insufficient (LOW) mice were assessed across a range of physical performance domains that include: rotarod latency to fall as the best 2 of 3 trials (**A**), and grip endurance as the best of two trials timed for latency to fall from a grid apparatus (**B**). Statistical significance indicated by "**" p< 0.01.
